# Bipolar disorder and maternity

**DOI:** 10.1192/j.eurpsy.2022.2225

**Published:** 2022-09-01

**Authors:** M. Palomo Monge, M.V. López Rodrigo, C. García Montero, A. Osca Oliver, M.F. Tascón Guerra, M. Pérez Fominaya, V. Ros Font

**Affiliations:** 1 Hospital Nuestra Señora del Prado, Psiquiatria, Talavera de la Reina, Spain; 2 Hospital Nuestra Señora del Prado, Psiquiatría, Talavera de la Reina, Spain; 3 Hospital Provincial de Ávila, Servicio De Psiquiatría, Ávila, Spain

**Keywords:** Pregnancy, bipolar disorder, woman and mental health, maternity

## Abstract

**Introduction:**

Bipolar disorder can be a severe psychiatric disorder. The combined prevalence of bipolar I, bipolar II, and unspecified bipolar disorders according to DSM-IV is 1.8%. Mean age at first affective episode has been estimated to 20 years among out-patients in the United States (2).

**Objectives:**

We present the case of a 40-year-old patient, diagnosed with type I bipolar disorder. In her story, multiple admissions are recorded for both manic and depressive episodes. The patient showed a desire to be a mother and multiple therapeutic interventions were performed, de-escalation of stabilizers until she was withdrawn, which triggered generally manic episodes that required hospital admission.

**Methods:**

Given the controversy in the decision to maintain or not drug treatment during pregnancy and the lack of clear criteria, in this case it was decided to try to gradually withdraw the treatment, which triggered several serious relapses. It was then decided to maintain the treatment at lower doses than usual or complete withdrawal, which in all cases precipitated relapses. Finally the patient reconsidered her wishes and abandoned the possibility of pregnancy.

**Results:**

Bipolar I Disorder

**Conclusions:**

Although most studies have found similar lifetime prevalence rates of bipolar disorder between men and women, gender differences may be evident in the impact of reproductive life events on affected women. In addition to the controversy regarding the decision to maintain or not treatment during pregnancy, there is also the certainty that childbirth can be the specific trigger for a manic or hypomanic episode.

**Disclosure:**

No significant relationships.

